# Deep Neural Network-Based Phase-Modulated Continuous-Wave LiDAR

**DOI:** 10.3390/s24051617

**Published:** 2024-03-01

**Authors:** Hao Zhang, Yubing Wang, Mingshi Zhang, Yue Song, Cheng Qiu, Yuxin Lei, Peng Jia, Lei Liang, Jianwei Zhang, Li Qin, Yongqiang Ning, Lijun Wang

**Affiliations:** 1 State Key Laboratory of Luminescence and Applications, Changchun Institute of Optics, Fine Mechanics and Physics, Chinese Academy of Sciences, Changchun 130033, China; 2 University of Chinese Academy of Sciences, Beijing 100049, China; 3 Peng Cheng Laboratory, Shenzhen 518055, China

**Keywords:** deep neural networks, lidar, phase-modulated continuous-wave, pulse width

## Abstract

LiDAR has high accuracy and resolution and is widely used in various fields. In particular, phase-modulated continuous-wave (PhMCW) LiDAR has merits such as low power, high precision, and no need for laser frequency modulation. However, with decreasing signal-to-noise ratio (SNR), the noise on the signal waveform becomes so severe that the current methods to extract the time-of-flight are no longer feasible. In this paper, a novel method that uses deep neural networks to measure the pulse width is proposed. The effects of distance resolution and SNR on the performance are explored. Recognition accuracy reaches 81.4% at a 0.1 m distance resolution and the SNR is as low as 2. We simulate a scene that contains a vehicle, a tree, a house, and a background located up to 6 m away. The reconstructed point cloud has good fidelity, the object contours are clear, and the features are restored. More precisely, the three distances are 4.73 cm, 6.00 cm, and 7.19 cm, respectively, showing that the performance of the proposed method is excellent. To the best of our knowledge, this is the first work that employs a neural network to directly process LiDAR signals and to extract their time-of-flight.

## 1. Introduction

Light detection and ranging (LiDAR) technology is an active detection and ranging system that uses lasers as the emitting light sources. Thanks to its short wavelength, directionality, and the monochromaticity of the lasers, LiDAR can achieve high-precision, high-range resolution, and high-angle resolution [1,2,3]. In turn, LiDAR has found applications in various fields, such as autonomous driving, drones, virtual reality, and space surveillance [4,5].

Common LiDAR ranging methods may be divided into two main classes: time-of-flight (ToF) and frequency-modulated continuous-wave (FMCW) schemes [1,2,3,6,7]. The ToF LiDARs are simple to implement. They are based on high-power pulsed light, which allows one to achieve long-distance measurement [1,2,3,6]. However, there is a potential eye-safety issue and ToF schemes lack the capability of velocity measurement. Additionally, their anti-interference capability is poor, which limits their use in future LiDAR applications [8]. LiDAR based on FMCW uses linear frequency modulation to mark time information on the frequency of the laser and demodulates the difference frequency signal through the coherent mixing with a local oscillator light and echo light [6]. Combined with the modulation rate, the detection distance is finally obtained, which has the advantages of low power consumption, long range, and high accuracy [9]. However, the current development of frequency modulation technology is immature, since the commonly used direct-modulated semiconductor lasers require iterative learning to improve the linearity of frequency modulation and are easily affected by environmental factors [10,11]. If single-sideband modulation is used, linearity is improved, yet the chip loss remains large such that the output power is low, leading to a limited detection range [12,13]. Moreover, the overall equipment is too expensive. In summary, both ToF and FMCW ranging methods are affected by various fundamental problems that are difficult to solve. On the other hand, with the rapid development of autonomous driving technology and the increasing demand for effective and affordable LiDARs, the need for novel LiDAR ranging methods is critical.

In order to resolve the aforementioned problems, our previous study proposed a novel ranging method called Phase-Modulated Continuous-Wave (PhMCW) [14]. This method involves modulating the phase of the laser (time coding) and using coherent mixing to accurately demodulate the flight time, thereby achieving ranging.

**Figure 1:**
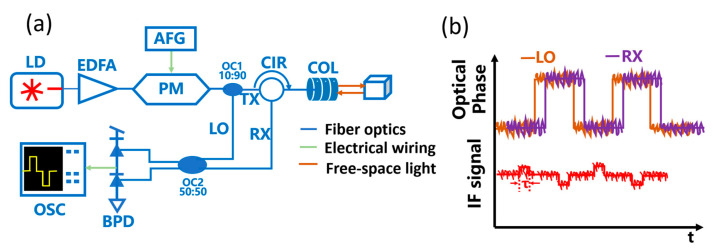
(**a**) Schematic of the PhMCW LiDAR. LD—Laser; COL—collimator; OSC—oscilloscope. (**b**) Optical phase of the LO and RX (**top panel**) and waveform of the IF signal (**bottom panel**).

The schematic diagram of the PhMCW setup is shown in Figure 1. The light source is a 1550 nm continuous-wave laser, equipped with an erbium-doped fiber amplifier (EDFA) to compensate for the internal optical loss of the system. The following stage is a phase modulator (PM), which is driven by an arbitrary function generator (AFG) to achieve rectangular wave modulation of the laser phase. The modulated laser is then split into a local oscillator beam (LO) and a detection beam (TX) by an optical coupler (OC). The TX beam is collimated and directed towards the target after passing through a circulator. The reflected beam (RX) is received by the collimator. After mixing with the coupler, the LO and the RX are converted into electrical signals by a balanced detector (BPD), at an intermediate frequency (IF). The IF signal is a continuous set of pulse signals, and the pulse width is equal to the flight time. According to the formula: *d* = (*c* × *τ*)/2, the detection distance *d* can finally be obtained.

The key stage of the PhMCW ranging method is the measurement of the pulse width of the IF signal. As long as the pulse width can be accurately measured, the distance can be accurately evaluated. The pulse width can be directly read in the time domain, e.g., by time digital conversion (TDC), digital counting, and high-frequency oscilloscope scanning [15]. They are based on detecting the rise and fall edges of the pulse and evaluating the pulse width as their difference. For the PhMCW scheme, the signal-to-noise ratio (SNR) is scaled as $SNR\propto \frac{\sqrt[{2}]{\rho }}{d}$, where *ρ* is the target reflectance and *d* is the distance [14]. As a consequence, the SNR becomes very low for large distances or low reflectivity objects. In those cases, the noise on the signal waveform becomes so severe that current methods to evaluate the pulse width are no longer feasible. Overall, this poses limitations to the detection distance achievable by PhMCW LiDARs.

In this paper, to overcome this difficulty, we propose using neural networks to measure pulse width, which can enhance the long-distance ranging ability of the PhMCW method and has significant implications for its application in several fields. This work focuses on the front-end stage and, for the first time, employs a neural network to analyze digitized IF signals and directly obtain the distance information rather than a post-processing point cloud. To the best of our knowledge, this is the first work that employs a neural network to directly process LiDAR signals and obtain the distance.

## 2. Analysis

**Figure 2:**
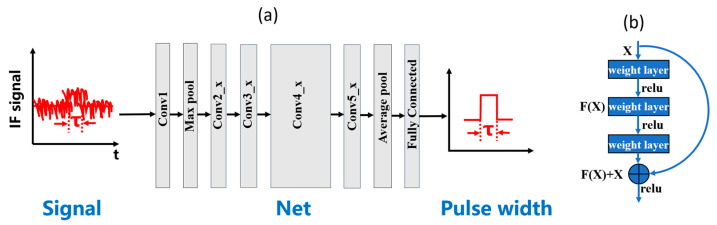
(**a**) Schematic of IF pulse width recognition network; (**b**) schematic of the residual structure.

Deep neural networks have powerful learning and computing capabilities and are widely used in image recognition and data analysis. Previous applications of neural networks in the field of LiDAR lie mainly in point cloud processing [16,17,18]. The post-processing of point clouds facilitates noise cancellation, object recognition, and so on. However, the distance of each point in the point cloud is extracted by conventional methods. For example, in ToF LiDARs, the distance is obtained by analyzing the rising and falling edges of the electrical signal and measuring their time differences. In this paper, the problem we are dealing with is to measure the pulse width by analyzing the time-domain waveform obtained by the PhMCW LiDAR, as shown in Figure 2a.

There are two approaches to this problem: one is using a regression network [19,20,21], which involves regressing the input waveform to obtain a regression model that minimizes the average error in predicting the pulse width of the input waveform. This method returns continuous values and allows one to obtain pulse widths with many significant digits (it even corrects to the first six decimal places). Such high resolution is far too high for actual LiDAR applications, where the general ranging resolution is within the range of 5–30 cm [22,23,24,25,26], and consequently pursuing results with such high resolution is meaningless.

Another approach is the use of classification techniques [27,28,29], which consider the pulse widths as a set of values with possible categories and assign a category to each input waveform. This method produces pulse widths within a discrete set of values and can stably measure pulse widths with a given resolution. This is exactly what is needed in real applications. Therefore, in this paper, we establish a reliable mapping between time-domain waveform and pulse width, in order to achieve the classification of the pulses.

Conventional networks used for such classification include Lenet, Alexnet, VGGnet, Googlenet, Resnet [30,31,32,33,34], etc. Compared to the other networks, Resnet has a larger network depth while maintaining a relatively low complexity. It establishes residual learning models and introduces residual structures, such that the network maintains high accuracy while increasing depth, ultimately establishing a network with sufficient depth to achieve high accuracy in large-scale classifications. As shown in Figure 2b, this structure has feedback between the input and the output, and by learning the difference between the input and the output, the loss of information transmission between the layers is minimized, thus achieving a deep network.

**Figure 3:**
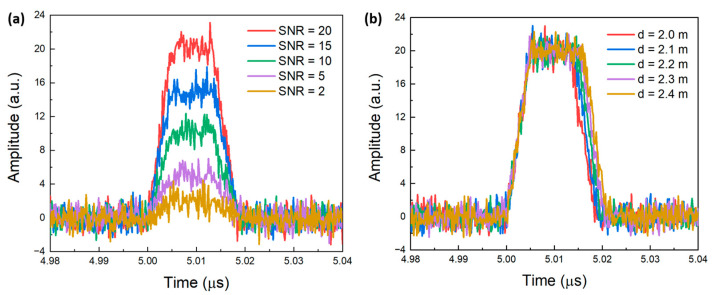
Waveforms at different SNRs (**a**) and distances (**b**).

In our classification task, there are two main challenges. The first one is that when the SNR is low, the pulse width information in the waveform becomes very blurry and difficult to obtain, as shown in Figure 3a. The second one is that when the resolution is high, the similarity between the waveforms is too high to distinguish the distance differences between the IF waveforms. From Figure 3b, when the distance is too close, such as *d* = 2.3 m or *d* = 2.4 m, the difference between the pulse widths is very trivial and is difficult to distinguish. Based on these observations, we need to design a network that effectively classifies items when the inter-class differences are low, and when the classification information is fuzzy. The solution to both these two points is to design a network with sufficient depth. Overall, these requirements can be satisfied by Resnet152 [34], which we will use in our experiment.

## 3. Experimental Section and Results

The experiment is divided into three steps. Firstly, we explore the influence of distance resolution on network recognition accuracy. Subsequently, the influence of SNR on network accuracy is discussed. Finally, we perform a proof-of-concept point cloud imaging demonstration.

**Figure 4:**
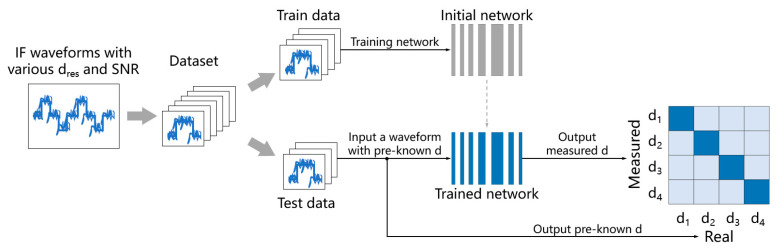
Process of analyzing influences of distance resolution and SNR on accuracies of the networks.

Figure 4 shows the experimental process. The training dataset is obtained using simulated IF waveforms with various pulse widths, distance resolutions, and signal-to-noise ratios. The training platform is TensorFlow 1.14 and Keras 2.25, and the optimizer used is the ADAM optimization algorithm. The training process is supervised learning, and the network converges through 60–100 iterations.

### 3.1. Influence of Resolution on Network Accuracy

First, we assess the resolution achievable by the network. Here, we define the resolution as the inter class difference in the neural network classification, i.e., the interval of distance difference. We set the SNR to be 20, which is relatively high and, therefore, effectively eliminates the influence of noise on the waveform pulse width. At such SNR, the influence of noise can be ignored and the recognition ability of the neural networks for time-domain waveform pulse width can be explored. The resolution is set to be from 0.5 m to 0.1 m. As a proof-of-concept demonstration, the number of classifications is set to be 30 to 90, which is large enough to demonstrate the validation of the proposed method but not so large that training the network becomes excessively time-consuming. The distance range is determined to be either from 1.8 m to 10.7 m or from 2.8 m to 20.7 m, which minimizes the number of variables meanwhile meeting the requirement of the distance resolution and classification quantity. Please note the maximal distance range is set to be 10.7 m and 20.7 m, respectively. This is because collecting datasets up to 20.7 m is not excessively time-consuming. Indeed, we can extend the maximum distance to 200 m but doing so will greatly increase our workload (data collection, network training, etc.), and the obtained experimental results will not be any different from the current setup. Therefore, extending the distance range to fully meet the requirements of various applications is beyond the scope of this paper. In addition, in order to ensure that the network training results are as balanced as possible, the number of each class should be as equal as possible. At the same time, the amount of training data should not be too small, which would lead to insufficient training results, nor too much, which would result in too long training time. In the end, we decided to set the number of each class between 100 and 200 groups. As for the testing dataset, we artificially set the quantity to be 10 for each distance. For the current experimental setup, it takes a CPU approximately 10 days to train the network, and 15 ms to measure the distance, which meets the requirements of real-time measurement. A GPU, an FPGA, or a photonic artificial intelligent chip would significantly accelerate the measurement time by tens and hundreds of times. The performances of the networks with various resolutions are summarized in Table 1.

**Figure 5:**
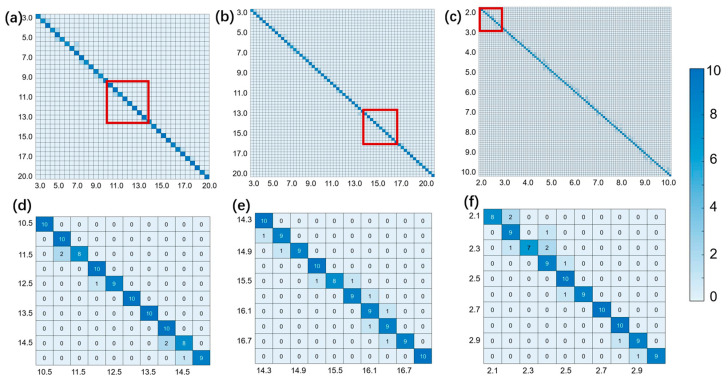
(**a**–**c**) Statistical matrixes of networks with resolutions of 0.5 m, 0.3 m, and 0.1 m, respectively; (**d**–**f**) Enlarged images of the red squared area in (**a**–**c**). Horizontal axis is real distance while vertical axis is measured distance.

The statistical matrixes of the test results are shown in Figure 5. The accuracy peaks at 97.8% at low resolutions of 0.5 m, while it decreases to 90.2% at high resolutions of 0.1 m. In the statistical matrix of the test results, as the resolution increases, more points deviate from the diagonal where the real and measured distances are equal. However, even for the highest resolution of 0.1 m, 90.2% of the measured distances are located at the diagonal, indicating the excellent performance of our method. Therefore, we can conclude that the network is able to achieve high accuracy at a high resolution of 0.1 m, which meets the requirements of most LiDAR applications.

### 3.2. Influence of SNR on Network Accuracy

We then explore the effect of SNR on the recognition ability of the network. We start from the previous experiment, where the resolution is 0.1 m and the SNR is 20, corresponding to a recognition accuracy of 90.2%. Next, we will gradually decrease the SNR and assess the network’s accuracy.

**Figure 6:**
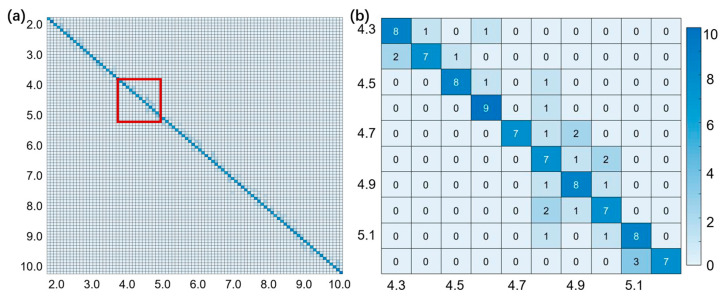
(**a**) Statistical matrix of network with a resolution of 0.1 m and a SNR of 2; (**b**) Enlarged image of the red squared area in (**a**). Horizontal axis is real distance while vertical axis is measured distance.

We take distances in a range from 1.8 m to 10.7 m, with 10,000 simulated waveforms for training and 900 waveforms for testing. The corresponding accuracies are summarized in Table 2. As the SNR decreases, the recognition accuracy of the network also decreases. For an SNR equal to 2, the recognition accuracy is down to 81.4%. If the SNR is lower, the accuracy will further decrease, leading to significant errors in the actual measurement, making this method inapplicable. Therefore, the lowest SNR and the highest resolution are 2 and 0.1 m, respectively, which corresponds to the strictest requirements in practical applications. The statistical matrix of the test results in this situation is shown in Figure 6.

**Figure 7:**
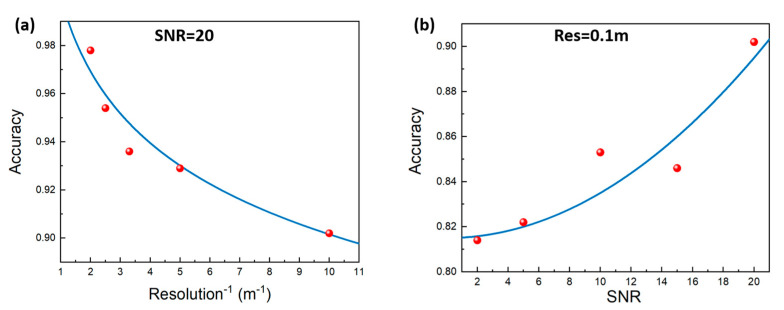
Accuracy as function of resolution (**a**) and SNR (**b**). The red dots represent experimental data points, while the blue lines represent curves fitted based on experimental data points.

In Figure 7, we show a scatter plot of the accuracy observed in different resolution and SNR experiments, together with the corresponding fitting curves. In Figure 7a, the accuracy scales as ACC = Resolution^−0.45^ in terms of the resolution, i.e., the accuracy gradually decreases with increasing resolution. This is because as the resolution increases, the difference in the pulse width intervals between the waveforms becomes smaller, which increases the difficulty for the network and leads to reduced accuracy. In Figure 7b, the accuracy is fitted by ACC = 0.0002 × SNR^2^ + 0.82 in terms of the SNR, i.e., the accuracy decreases quadratically with decreasing SNR. This is because the SNR affects the degree of interference of noise on the waveform. When the SNR is high, noise has almost no effect on the waveform, and the pulse width is clearly visible. However, when the SNR is low, noise has a significant impact on the waveform, and the pulse width is almost invisible due to the severe interference. This brings serious interference into the network for identifying the pulse width, and the accuracy will naturally be relatively low.

As shown in Figure 2b, one of the core structures of the network is the residual structure, which adds a skip branch between the data input and output. The output is obtained by summing the input and the input processed by the hierarchy; therefore, the data output will be affected by the input twice. In our experiment, when the decrease in SNR (or resolution) causes a change in the network input, it will be transmitted multiple times by the residual structure. In this way, the calculation of the parameters within the network will be fed back multiple times, and the final output result of the network will inevitably change more dramatically compared with the input change. Therefore, the accuracy will decrease faster, resulting in a nonlinear relationship between the SNR (or resolution) and the accuracy.

### 3.3. Point Cloud Imaging Demonstration

At this point, we built a neural network to directly process IF signal can accurately extract the distance. Using this network, we can achieve an SNR of 2, a resolution of 0.1 m, and a detection range of 1.8 m to 10.7 m. Using these results, we then move on to the proof-of-concept point cloud imaging experiments.

**Figure 8:**
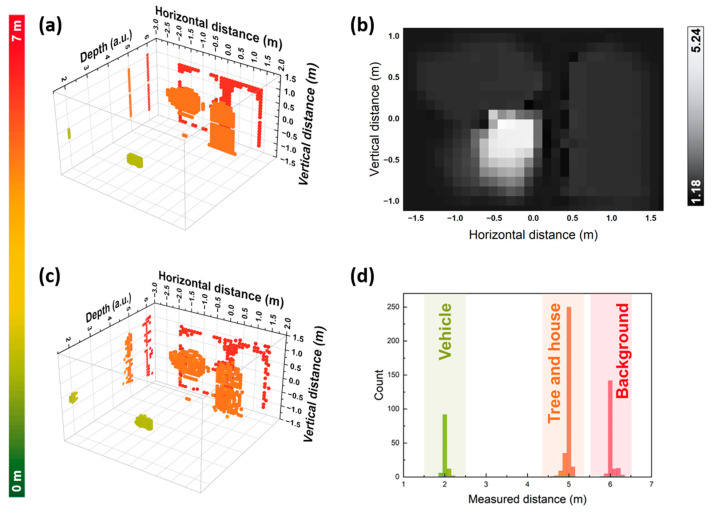
Point cloud imaging results: (**a**,**c**) are the original and the reconstructed point clouds with projections on the YZ plane, respectively; (**b**) SNR map of point cloud; (**d**) histogram of measured distances.

As shown in Figure 8a, we build a point cloud simulation platform that contains a vehicle located at 2 m, a tree and a house located at 5 m, and a background located at 6 m, respectively. The horizontal and vertical field-of-view of the scene are ±15° and ±10°, respectively. The angular resolution is 1°, resulting in a lateral resolution of 30 × 20. By adjusting the laser power and aperture of the optics, the SNR is calculated according to the LiDAR equation [35] and is in the range of 1.64 to 5.00, as shown in Figure 8b. Waveforms for each point are simulated according to the distance and the calculated SNR. Please note the resulting SNR is rather low and thus represents the worst-case performance of our method. In addition, the neural network is trained by a dataset with a specific SNR of 2, which is not the same as the SNR in the scene which ranges from 1.64 to 5.00, and may result in recognition error. We then feed these 600 IF waveforms into the neural network to obtain 600 predicted distance values and, in turn, 600 pieces of 3D point cloud data to reconstruct the scene. Figure 8c shows the reconstructed point cloud. Compared with the original scenario, the reconstructed point cloud has good fidelity, the object contours are clear, and the features of the objects are well restored. Figure 8d shows the distribution of the measured distances. The precision of the vehicle, the tree and the house, and the background are 4.73 cm, 6.00 cm, and 7.19 cm, respectively, which are comparable to commercial LiDAR products, suggesting promising applications.

## 4. Conclusions

In this paper, we explored the ability of neural networks to determine the pulse widths of LiDAR echoes, demonstrating that they can be reliably used to achieve the task. To the best of our knowledge, this is the first work that employs neural networks to directly process digitized LiDAR signals and to extract their time-of-flight. The recognition accuracy reaches 81.4% at a 0.1 m distance resolution with a SNR as low as 2. We simulate a scene that contains a vehicle located at 2 m, a tree and a house located at 5 m, and a background located at 6 m, respectively. The reconstructed point cloud has good fidelity, the object contours are clear, and the features of the objects are restored. The precision of the three objects are 4.73 cm, 6.00 cm, and 7.19 cm, respectively, showing that the performance of the proposed method is excellent and meets the requirements of practical applications. Overall, our results show that neural networks are an effective tool for measuring the pulse widths of PhMCW LiDAR time-domain waveforms.

## Figures and Tables

**Table 1:** Network performance at different resolutions.

Resolution (m)	Distance Range (m)	No. of Class	Total Training Quantity	Testing Quantity	Accuracy %
0.5	2.8–20.7	36	4000	360	97.8
0.4	2.8–20.7	45	5000	450	95.4
0.3	2.8–20.7	60	6000	600	93.6
0.2	1.8–10.7	45	5000	450	92.9
0.1	1.8–10.7	90	10,000	900	90.2

**Table 2:** Network performance under different SNRs.

Signal-to-Noise Ratio	Accuracy %
20	90.2
15	84.6
10	85.3
5	82.2
2	81.4

## Data Availability

The data presented in this study are available on request from the corresponding author.
